# Peritumoral vascular invasion and NHERF1 expression define an immunophenotype of grade 2 invasive breast cancer associated with poor prognosis

**DOI:** 10.1186/1471-2407-12-106

**Published:** 2012-03-22

**Authors:** Andrea Malfettone, Concetta Saponaro, Angelo Paradiso, Giovanni Simone, Annita Mangia

**Affiliations:** 1 Functional Biomorphology Laboratory, National Cancer Centre, Viale Orazio Flacco, 65-70124 Bari, Italy; 2 Scientific Direction, National Cancer Centre, Viale Orazio Flacco, 65-70124 Bari, Italy; 3 Pathology Department, National Cancer Centre, Viale Orazio Flacco, 65-70124 Bari, Italy

**Keywords:** NHERF1, Peritumoral Vascular Invasion, Histological grade, Breast cancer, VEGFR1, Nottingham Prognostic Index

## Abstract

**Background:**

Traditional determinants proven to be of prognostic importance in breast cancer include the TNM staging, histological grade, proliferative activity, hormone receptor status and HER2 overexpression. One of the limitations of the histological grading scheme is that a high percentage of breast cancers are still classified as grade 2, a category with ambiguous clinical significance. The aim of this study was to best characterize tumors scored as grade 2.

**Methods:**

We investigated traditional prognostic factors and a panel of tumor markers not used in routine diagnosis, such as NHERF1, VEGFR1, HIF-1α and TWIST1, in 187 primary invasive breast cancers by immunohistochemistry, stratifying patients into good and poor prognostic groups by the Nottingham Prognostic Index.

**Results:**

Grade 2 subgroup analysis showed that the PVI (p = 0.023) and the loss of membranous NHERF1 (p = 0.028) were adverse prognostic factors. Relevantly, 72% of grade 2 tumors were associated to PVI+/membranous NHERF1- expression phenotype, characterizing an adverse prognosis (p = 0.000). Multivariate logistic regression analysis in the whole series revealed poor prognosis correlated with PVI and MIB1 (p = 0.000 and p = 0.001, respectively). Furthermore, in the whole series of breast cancers we found cytoplasmic NHERF1 expression positively correlated to VEGFR1 (*r *= 0.382, p = 0.000), and in VEGFR1-overexpressing tumors the oncogenic receptor co-localized with NHERF1 at cytoplasmic level.

**Conclusions:**

The PVI+/membranous NHERF1- expression phenotype identifies a category of grade 2 tumors with the worst prognosis, including patient subgroup with a family history of breast cancer. These observations support the idea of the PVI+/membranous NHERF1- expression immunophenotype as a useful marker, which could improve the accuracy of predicting clinical outcome in grade 2 tumors.

## Background

Breast cancer represents a heterogeneous disease with an intrinsic complexity in cellular-biomolecular profile and in its responsiveness to treatment [1]. The management of early-stage breast cancer is based on clinical and pathological parameters which are able to predict distinct patient outcomes. Traditional determinants proven to be of prognostic importance and used in routine practice include the pathological subtype, TNM staging information, histological grade, proliferative activity, receptor status and human epidermal growth factor receptor 2 (HER2) overexpression.

The degree of histological differentiation in operable breast carcinomas has long represented one of the best established prognostic factors which have been validated in multiple independent studies [2-4]. The Elston and Ellis modification of the Scarff-Bloom-Richardson grading system separates breast cancer patients into distinct prognosis groups: grade 1, 2 or 3, with a low, intermediate or high risk of recurrence, respectively [5]. Although internationally accepted among pathologists, one of the limitations of the histological grading scheme is that a high percentage (30% to 60%) of breast cancer is still classified as grade 2, a category with ambiguous clinical significance [6].

To be of clinical use, a prognostic factor must show a wide separation in the outcome of the groups identified and select adequate numbers in each group [7]. Notwithstanding many efforts, no single prognostic factor in breast cancer meets these criteria. Histological grading has been combined with tumor size and lymph node stage to form the Nottingham Prognostic Index (NPI), which allows stratification of patients into three different prognostic groups [3] and satisfying these criteria.

Within the last decade, several attempts have been performed to classify grade 2 tumors into two distinct molecular subclasses, improving biological and clinical usefulness of histological grading [8-15]. A multitude of factors, such as HER2, p53, proliferation markers and vascular channel invasion, has been extensively studied and tested in clinical settings, but their importance needs to be validated in statistically robust studies.

Over the last years, laboratory research has proposed novel prognostic markers but not sufficiently investigated to demonstrate their prognostic value. Most of these markers are involved in breast cancer biology and related to essential aspects of cell life, proliferation, transformation and apoptosis [16]. Recently, we have demonstrated that the Na^+^⁄H^+ ^exchanger regulatory factor 1 (NHERF1), an adaptor protein for membrane macromolecular complexes, is a potential candidate of clinical relevance for human breast cancer [17-19]. The strong correlation with poor vascularization and the hypoxia-inducible factor-1α (HIF-1α), a marker of hypoxic tumors, indicates that NHERF1 expression might play an important role in driving metastatic progression by modifying the tumor microenvironment [20]. Among several hypoxia related genes, the vascular endothelial growth factor (VEGF) mediates its effects on proliferation and survival mostly through the VEGF receptor 1 (VEGFR1) and 2 (VEGFR2) within endothelial cells. Nevertheless, it has been demonstrated that VEGFR1 expression in breast cancer cells correlated significantly with high metastasis risk and relapse [21,22]. During malignant cancer progression, TWIST1 plays a role in the development of distant metastasis by inducing an epithelial-to-mesenchymal transition of epithelial breast cancer cells and by prompting them to enter the bloodstream [23,24].

In this study we examined traditional prognostic factors and a panel of protein markers associated with breast cancer progression, aggressiveness, hypoxic response and cell invasion/metastasis, respectively NHERF1, VEGFR1, HIF-1α and TWIST1, to determine whether they are differentially expressed in tumors scored as grade 2, trying to improve their prognosis definition.

## Methods

### Patients and tumor specimens

A selected series of 187 primary invasive breast carcinomas were included in this study: 48% (n = 90) were sporadic patients and 52% (n = 97) were classified as having a family history, after a genetic counseling program as reported previously [25]. None of the sporadic patients had a family history of breast or ovarian cancer. Table 1 lists the patients' clinicopathological features. All histological sections of tumor specimens were re-evaluated by two experienced pathologists, performed without any knowledge of patient history. Each patient was staged according to the International Union Against Cancer TNM classification [26]. The tumor size was ≤ 2 cm in 41% (n = 77) of cases and > 2 cm in 59% (n = 110) of cases. Pathological examination revealed that axillary lymph node status was positive in 59% (n = 101) and negative in 41% (n = 71) of patients. Histological grading was performed according to the Elston and Ellis method [2] and 17% (n = 31) of tumors were histological grade 1, 46% (n = 87) were grade 2 and 37% (n = 69) were grade 3. Moreover, within the familial cancer group, 49% (48/97) were grade 2. Of all tumors, 150 (80%) cases were invasive ductal carcinoma (IDC) not otherwise specified (NOS), with the remainder 37 (20%) consisting of other histological types including medullary, tubular, atypical medullary and lobular tumors. Assessment of the peritumoral vascular invasion (PVI) was based upon examination of sections stained with haematoxylin and eosin and was considered evident if at least one cohesive clump of tumor cells was clearly visible within peritumoral endothelial-lined spaces, both lymphatic channels and small blood vessels--closely associated with primary invasive carcinoma [27].

**Table 1:** Clinicopathological features and tumor marker expressions in a cohort of 187 invasive breast cancer patients

Parameter	Total, n	(%)
Age at diagnosis		
Median (range)	50 years	(24-83)
Tumor size		
≤ 2 cm	77	(41)
> 2 cm	110	(59)
Nodal status		
Negative	71	(41)
Positive	101	(59)
Tumor grade		
1	31	(17)
2	87	(46)
3	69	(37)
Histologic tumor type		
IDC (NOS)	150	(80)
Other histologic type	37	(20)
PVI		
Absent	114	(66)
Present	59	(34)
NPI		
Good (≤ 3.4)	49	(26)
Moderate (3.4-5.4)	88	(47)
Poor (> 5.4)	50	(27)
ER status		
Negative (≤ 10%)	53	(29)
Positive (> 10%)	131	(71)
PR status		
Negative (≤ 10%)	76	(42)
Positive (> 10%)	107	(58)
MIB1		
Negative (≤ 20%)	79	(43)
Positive (> 20%)	104	(57)
HER2 status		
Negative	133	(85)
Positive	23	(15)
Cytoplasmic NHERF1		
Negative (≤ 40%)	105	(69)
Positive (> 40%)	47	(31)
Membranous NHERF1		
Negative (0%)	134	(87)
Positive (> 0%)	20	(13)
VEGFR1		
Negative (≤ 2%)	82	(50)
Positive (> 2%)	82	(50)
HIF-1α		
Negative (0%)	98	(63)
Positive (> 0%)	58	(37)
TWIST1		
Negative (≤ 3%)	69	(49)
Positive (> 3%)	73	(51)

*Abbreviations*: Tumor grade = histological differentiation grade;

IDC (NOS) invasive ductal carcinoma (not otherwise specified);

PVI = peritumoral vascular invasion;

NPI = Nottingham Prognostic Index;

ER = estrogen receptor; PR = progesterone receptor;

HER2 = human epidermal growth factor receptor 2

The NPI was calculated according to the following equation: NPI = tumor size (cm) × 0.2 + tumor grade (1-3) + lymph node stage (A-C). Stage A, stage B and stage C denoted lesions with 0, with ≤ 3 and with > 3 involved lymph nodes, respectively [28]. Thus, on the basis of obtained NPI, each patient was assigned to one of three prognostic groups: Good (NPI ≤ 3.4), Moderate (3.4 < NPI ≤ 5.4) and Poor (NPI > 5.4). Then, we verified whether breast cancers with Good, Moderate or Poor prognosis were associated with a series of well-defined biological factors and with tumor markers not currently used in routine diagnosis.

Variables evaluated for the expression analysis were: estrogen receptor (ER) and progesterone receptor (PR) status, tumor proliferative activity (MIB1), HER2 status, NHERF1, VEGFR1, HIF-1α and TWIST1.

Information regarding patient characteristics, including age, tumor size, nodal status, tumor grade, histologic tumor type, PVI, MIB1, ER, PR and HER2 status, was collected from the Pathology Department of our Institute. ER, PR and MIB1 immunostainings were confined to the nucleus and were performed according to method previously explained [25]. ER and PR were regarded overexpressed when > 10% of nuclei were positive. Cases with a MIB1 index > 20% were considered high proliferating tumors. The MIB1 cut-off represents the median value of the scores relative to all breast tumor samples analyzed during the last five years within our Institute. The HER2 status was scored as 0, 1+, 2+ or 3+, using a monoclonal antibody (MoAb clone CB11, Novocastra Laboratories Ltd, Newcastle, UK), in accordance with the Herceptest scoring system (Food and Drug Administration accepted): 0, no membranous immunoreactivity or < 10% of cells reactive; 1+, incomplete membranous reactivity in > 10% of cells; 2+, > 10% of cells with weak to moderate complete membranous reactivity; and 3+, strong and complete membranous reactivity in > 10% of cells. Cytoplasmic immunoreactivity was ignored. Cases scored 0 and 1+ were classified as negative, and cases scored 3+ were classified as positive. Cases regarded as indeterminate (2+) were tested for HER2 gene amplification by fluorescence in situ hybridization (FISH), as previously reported [17]. Briefly, using a dual probe system of different colors (PathVysion HER-2 DNA probe kit, Vysis-Olympus, Milan, Italy), the gene copy numbers of HER2 and centromeres of the corresponding chromosome 17 were retrieved. The FISH results were regarded as positive when the HER2/CEP17 ratio was ≥ 2.2. Cases with ratio 1.8 and 2.1 were defined as borderline. A signal was defined as significantly amplified if it was overrepresented in approximately 20% of nuclei. The study was performed with the approval of the Ethics Committee of our Institute. Each individual involved in the study signed an informed consent form authorizing the Institute to utilize their biological tissues for research purpose. All the analyses were performed in the ISO9001-2000 certified Clinical Experimental Oncology Laboratory of the National Cancer Centre of Bari (DNV Certificate No CERT-17885-2006-AQ-BRI-SINCERT).

### Immunohistochemistry

Sections of 4 μm-thickness were immunohistochemically stained using standard immunoperoxidase techniques. Slides were deparaffinized and rehydrated through a graded ethanol series. Endogenous peroxidase activity was blocked by incubation in 0.3% H_2_O_2 _buffer solution. Epitope antigen retrieval was carried out by boiling of slides in 0.01 M sodium citrate buffer (pH 6.0). For NHERF1, sections were incubated with a rabbit polyclonal EBP50 antibody (PA1-090, 1:150 dilution; Affinity Bioreagents, Golden, CO, USA). Immunohistochemical analysis of NHERF1 was based on subcellular localization of the protein and classified as cytoplasmic and/or membranous for each sample, as previously described [17]. According to median value cut-off, cases were classified positive when cytoplasmic NHERF1 immunoreactivity was present in > 40% of tumor cells and when membranous NHERF1 expression was detected in > 0% of tumor cells examined. HIF-1α staining was performed with a rabbit polyclonal antibody (H206, 1:100 dilution; Santa Cruz Biotechnology Inc., Santa Cruz, CA, USA). For HIF-1α only cells with completely and darkly stained epithelial nuclei were regarded as positive. HIF-1α was considered overexpressed when > 0% of nuclei were positive and the typical expression pattern (perinecrotic or diffuse) was noted [29]. Cytoplasmic staining of HIF-1α, observed occasionally, was ignored. The rabbit polyclonal antibody anti-VEGFR1 (C-17, 1:100 dilution; Santa Cruz Biotechnology Inc.), which recognizes the C-terminus of the human receptor 1 for VEGF, was incubated for 1 h at room temperature [30]. VEGFR1 was mainly observed in the cytoplasm of breast cancer tissues, and the staining was evaluated as percentage of immunoreactive cells. The cases were classified positive when VEGFR1 immunoreactivity was > 2% of tumor cells examined. For TWIST1 analysis, the sections were incubated with a mouse monoclonal antibody (Twist2C1a, 1:50 dilution; Abcam, Cambridge, United Kingdom) and only cells with > 3% of stained epithelial nuclei were regarded as positive, according to median value.

All immunohistochemically stained samples were scored in a blind manner by two independent observers. Protein expression was quantified by counting the positive cells in 3 representative areas for each section, and expressed as percentage of positive cells/section. Whether a section was uninformative, either lost or contained no tumor tissue, a case was judged as 'not evaluable' in the statistical analysis.

### Immunohistofluorescence

Immunofluorescent analysis was performed as described previously [17,31]. Briefly, formalin-fixed and paraffin embedded tissue serial sections of 3 μm in thickness were deparaffinized with xylene, and rehydrated in an ethanol series. Antigen retrieval was carried out immersing slides in a 0.01 M saline citrated buffer (pH 6.0) at 95°C for 40 min, then tissues were permeabilized with 0.1% Triton X100-Phosphate Buffered Saline for 15 minutes, blocked 30 min with 1% Bovine Serum Albumin-Phosphate Buffered Saline and incubated overnight at 4°C in a humidified chamber or with mouse monoclonal antibody anti-NHERF1 (6/EBP50, 1:20 dilution; BD Transduction Laboratories, Franklin Lakes, NJ, USA) together with a rabbit polyclonal antibody anti-VEGFR1 (C-17, 1:40 dilution; Santa Cruz Biotechnology Inc.) or with a mouse monoclonal anti-CD31 (NCL-CD31-1A10, 1:50 dilution; Novocastra Laboratories Ltd.) together with a rabbit polyclonal anti-NHERF1 (PA1-090, 1 μg/100 μL dilution; Affinity Bio-Reagents). The slides were then incubated at room temperature for 1 h with the Alexa Fluor 488 and Alexa Fluor 568 immunoglobulin G secondary conjugated antibodies (1:2000 dilution; Molecular Probes Inc., Eugene, OR, USA) and mounted with DAPI (ProLong^® ^Gold antifade reagent; Molecular Probes Inc.). Positive control slides that were run simultaneously were used for assessing the quality of immunoreactivity. For negative controls, slide sections that were immunopositive were treated with 1% Bovine Serum Albumin instead of the primary antibody, and no reactivity was observed in any of these controls. Images were obtained on a BX40 microscope (Olympus, Tokyo, Japan) with a SenSys 1401E-Photometrics charge-coupled device camera. To verify protein colocalization, each acquired stack was merged by transforming the three channels corresponding to red (tetramethylrhodamine B isothiocyanate), green (fluorescein isothiocyanate) and blue (4',6-diamidino-2-phenylindole) into a single three-color stack by using the "RGB merge" command of ImageJ software (National Institutes of Health Bethesda, MD).

### Statistical analysis

Analysis of tumor marker expressions and various clinicopathological features was determined by the Fisher's exact, Pearson *χ*^2 ^or *χ*^2 ^test as appropriate. Correlation between two continuous variables was assessed by the Pearson's rank test. A multivariate logistic regression model was used for multivariate analysis, computing odds ratio (OR) and 95% confidence intervals (95% CI). All tests were two-sided with a 95% CI, and a p value less than 0.05 was considered statistically significant. Data analysis was carried out using the statistical package SPSS 15.0 (SPSS Inc., Chicago, IL, USA).

## Results

### Relationship between tumor markers and clinicopathological features

A summary of the tumor marker expressions categorized according to the median values is provided in Table 1. Overexpression of cytoplasmic and membranous NHERF1, VEGFR1, HIF-1α and TWIST1 was shown in 31%, 13%, 50%, 37% and 51% of tumors, respectively.

**Figure 1:**
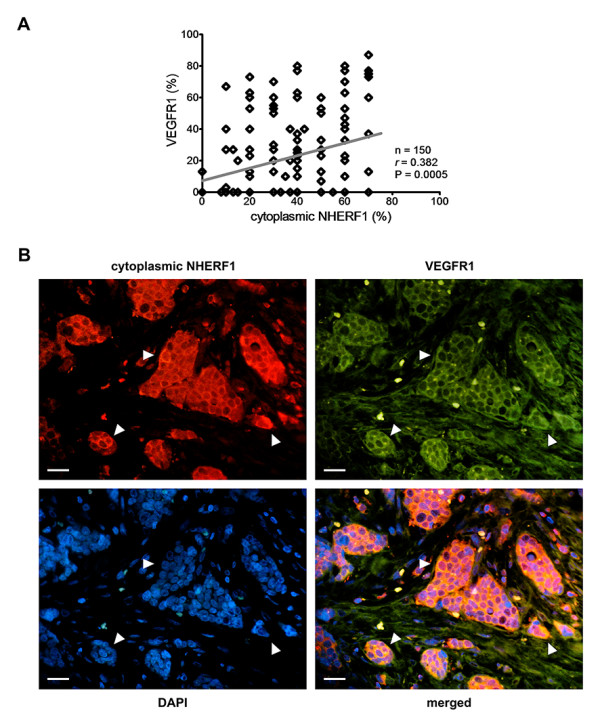
**Analysis of cytoplasmic NHERF1 and VEGFR1 expressions in invasive breast cancer**. (A) The correlation between protein expression of cytoplasmic NHERF1 and VEGFR1 was evaluated by Pearson's rank correlation coefficient analysis, and a positive significant correlation was established. (B) A representative tissue sample stained with NHERF1 and VEGFR1 antibodies and detected with Alexa Fluor 568 (red) and Alexa Fluor 488 (green) secondary antibodies, respectively, prior to fluorescence microscopy analysis. Arrowheads indicate invasive cells disseminated into the stroma with a high global expression of two proteins, where NHERF1 co-localized with VEGFR1 on cytoplasmic and membranous compartments. Scale bar = 10 μm.

The significant associations between tumor markers and clinicopathological features are summarized in Table 2. All breast cancers showed NHERF1 protein localized in the cytoplasm of tumor cells and 31% of overexpressing cytoplasmic NHERF1 tumors exhibited a significant association with tumor grade 3 (p = 0.035), negative PR status (p = 0.008), high MIB1 (p = 0.033), positive HER2 status (p = 0.036) and with moderate NPI (p = 0.029). In 13% of tumors, in addition to cytoplasmic NHERF1 immunoreactivity, NHERF1 showed also a plasma membrane localization; these overexpressing membranous NHERF1 tumors (13%) were significantly associated with tumor grade 2 (p = 0.037), positive PR status (p = 0.031), low MIB1 (p = 0.029) and with good NPI (p = 0.016). HIF-1α expression showed a significant association with tumor grade 2 (p = 0.001), negative PR status (p = 0.013), high MIB1 (p = 0.020) and moderate NPI (p = 0.029). VEGFR1 expression showed a significant association with negative PR status (p = 0.006). Expression of TWIST1 was statistically prevalent in low MIB1 tumors (p = 0.040). There was no statistically significant association between NHERF1, VEGFR1, HIF-1α, TWIST1 expressions and age, tumor size, PVI, nodal and ER status. Of 46 tumors with positive cytoplasmic NHERF1 expression, 31 (67%) exhibited positive VEGFR1 expression. While, of 104 cases with negative cytoplasmic NHERF1, 54 (52%) exhibited negative VEGFR1 expression. Pearson's rank test showed that cytoplasmic NHERF1 expression was positively correlated to VEGFR1 (*r *= 0.382, p = 0.000) (Figure 1A). The analysis of their relative localization by immunohistofluorescence studies indicated that the receptor VEGFR1 colocalized with NHERF1 when both proteins were overexpressed within cytoplasmic and/or membranous compartments in invasive clusters disseminated into the stroma (Figure 1B). Tumors positive for both cytoplasmic NHERF1 and VEGFR1 expressions, compared to those with negative expressions, resulted significantly associated with tumor grade 3 (p = 0.006), negative ER (p = 0.045) and PR status (p = 0.000) (data not shown). Furthermore, correlation analysis revealed that cytoplasmic NHERF1 expression levels were positively correlated with increasing ER levels (p = 0.022) (data not shown).

**Table 2:** Association between tumor markers expression and clinicopathological features

Parameter	Cytoplasmic NHERF1	Membranous NHERF1	HIF-1α	VEGFR1	TWIST1
	**Positive**	**Positive**	**Positive**	**Positive**	**Positive**
Tumor grade					
1	6 (21)	7 (25)	13 (48)	12 (44)	15 (58)
2	17 (24)	14 (19)	33 (49)	34 (46)	35 (57)
3	23 (43)	3 (6)	12 (20)	36 (57)	23 (43)
p value^1^	0.035	0.037	0.001	NS	NS
PR status					
Negative	27 (43)	4 (6)	29 (44)	43 (63)	29 (45)
Positive	19 (22)	16 (18)	18 (24)	37 (40)	42 (56)
p value^2^	0.008	0.031	0.013	0.006	NS
MIB1					
Negative	14 (22)	13 (21)	20 (28)	30 (44)	38 (61)
Positive	34 (40)	7 (8)	37 (46)	50 (54)	33 (43)
p value^2^	0.033	0.029	0.020	NS	0.040
HER2 status					
Negative	34 (29)	15 (12)	46 (37)	67 (53)	55 (49)
Positive	11 (55)	2 (11)	8 (40)	11 (55)	14 (70)
p value^2^	0.036	NS	NS	NS	NS
NPI					
Good	5 (13)	10 (26)	10 (24)	18 (45)	21 (58)
Moderate	24 (32)	8 (11)	28 (37)	40 (50)	33 (49)
Poor	15 (38)	2 (5)	20 (53)	24 (55)	19 (49)
p value^1^	0.029	0.016	0.029	NS	NS

Data presented as number of tumors and (%)

*Abbreviations*: Tumor grade = histological differentiation grade; PR = progesterone receptor; HER2 = human epidermal growth factor receptor 2; NPI = Nottingham Prognostic Index.

^1 ^p values were calculated with the use of the *χ*^2 ^test;

^2 ^p values were calculated with the use of the Fisher's exact test

Out of 187 cancers, 87 (46%) were grade 2 (Table 1) and these statistically inversely correlated with the PVI (p = 0.000), tumor size (p = 0.036), nodal status (p = 0.012) and MIB1 (p = 0.000), and directly correlated with ER (p = 0.000) and PR (p = 0.025) status.

### Prognosis analysis

**Figure 2:**
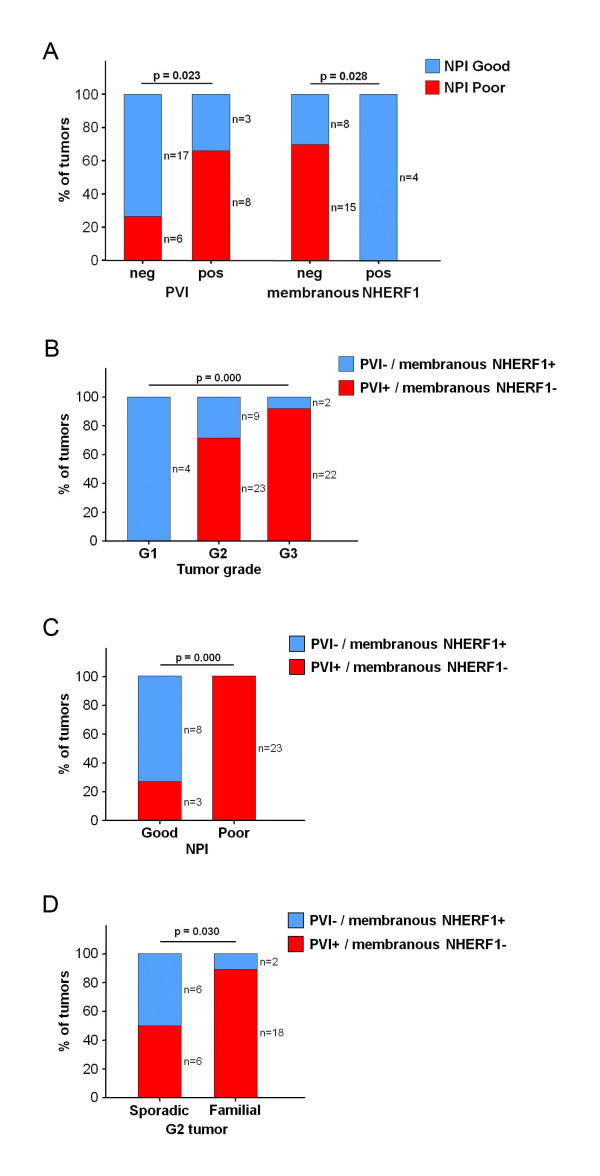
**Prognostic relevance of peritumoral vascular invasion and membranous NHERF1 in invasive breast cancer**. (A) Subgroup analysis revealed that the presence of PVI and the loss of membranous NHERF1 expression were adverse prognostic factors for grade 2 tumors (by Fisher's exact test). (B) The distribution analysis in the three distinct histological groups showed the PVI+/membranous NHERF1- expression phenotype significantly associated both to grade 2 and to grade 3 tumors (by *χ*^2 ^test). (C) The PVI+/membranous NHERF1- expression immunophenotype predicted poor prognosis in the whole cohort (by Fisher's exact test). (D) The PVI+/membranous NHERF1- expression immunophenotype correlated significantly with poor clinical outcome also in the subgroup of grade 2 familial tumors (by Fisher's exact test). *Abbreviations*: NPI = Nottingham Prognostic Index; PVI = peritumoral vascular invasion; neg = negative; pos = positive.

**Figure 3:**
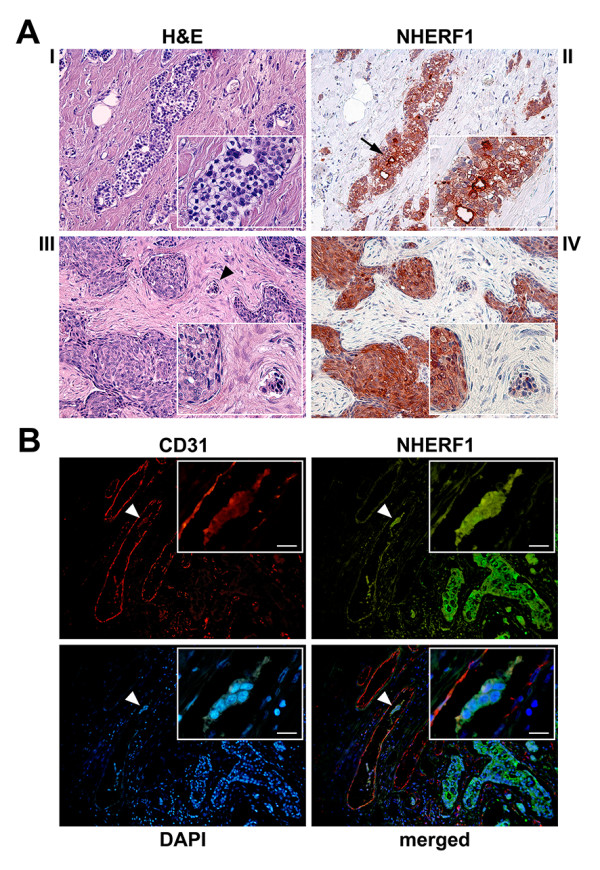
**Peritumoral vascular invasion and NHERF1 expression in grade 2 invasive breast carcinoma**. (A) Representative images of peritumoral vascular invasion by H&E and NHERF1 protein expression by immunoistochemistry: (I) a tumor with the absence of peritumoral vascular invasion and (II) with the overexpression of membranous NHERF1, in addition to cytoplasmic localization (arrow). (III) A case showing peritumoral vascular invasion (arrowhead) and (IV) negative expression of membranous NHERF1. Original magnification × 100, inset × 200. (B) Assessment of the peritumoral vascular invasion in a breast tumor section stained with CD31 and NHERF1 antibodies and detected with Alexa Fluor 568 (red) and Alexa Fluor 488 (green) secondary antibodies, respectively. Immunofluorescence analysyis shows a tumor cell cluster within the endothelial-lined vascular space (arrowheads), with strong cytoplasmic NHERF1 expression similarly to the invasive cellular component at right zone of the image. Scale bar = 10 μm.

When we applied the NPI to 187 breast patients, 49 (26%) were in the good prognostic group, 88 (47%) in the moderate prognostic group and 50 (27%) in the poor prognostic group (Table 1). We examined if tumors with grade 2 and poor prognosis were associated with some distinct clinicopathological parameters or with some tumor markers not currently used in routine diagnosis. Subgroup analysis revealed that the PVI (p = 0.023) and negative membranous NHERF1 expression (p = 0.028) were adverse prognostic factors for grade 2 tumors (Figure 2A, 3). When we analyzed the distribution of the PVI/membranous NHERF1 immunophenotypes in the three distinct histological groups, we showed that 72% of grade 2 and 92% of grade 3 tumors were significantly associated to the PVI+/membranous NHERF1-expression phenotype, both characterized by a poor prognosis (p = 0.000) (Figure 2B). Then, we explored the prognostic relevance of the PVI/membranous NHERF1 immunophenotypes in the whole cohort and, notably, 100% of tumors with poor prognosis significantly displayed the PVI+/membranous NHERF1- expression phenotype, compared with 27% of tumors with good prognosis (p = 0.000) (Figure 2C). Moreover, the PVI+/membranous NHERF1-phenotype in the subgroup of grade 2 familial tumors showed a higher significant proportion than proportion within subgroup of grade 2 sporadic tumors (90% vs. 50%; p = 0.030) (Figure 2D).

Univariate and binary logistic regression analyses on 187 cancers were performed to evaluate the associations between tumor markers and NPI. The tumor size, PVI, ER status, expression of MIB1 and HIF-1α were all significant factors strongly associated with worse prognosis (p = 0.031, p = 0.000, p = 0.000, p = 0.001 and p = 0.011, respectively). Interestingly, only the presence of membranous NHERF1 resulted a favorable prognostic factor (p = 0.012) (data not shown). Subsequently, when we have evaluated the prognostic relevance of the significant univariate parameters, a multivariate logistic regression analysis revealed poor prognosis correlated with the PVI and MIB1 (p = 0.000 and p = 0.001, respectively) (Table 3).

**Table 3:** Multivariate logistic regression analysis for tumor markers predicting prognosis of 187 invasive breast cancer patients

Variable	Odds ratio	95% CI	p value
PVI	8.656	6.186 - 9.862	0.000
Negative vs Positive			
ER status	0.007	0.000 - 0.013	0.130
Negative vs Positive			
MIB1	6.130	4.160 - 6.995	0.001
Negative vs Positive			
Membranous NHERF1	1.185	0.624 - 1.830	0.154
Negative vs Positive			
HIF-1α	1.392	0.458 - 1.865	0.178
Negative vs Positive			

*Abbreviations*: PVI = peritumoral vascular invasion; ER = estrogen receptor

## Discussion

Clinical and pathological factors, such as nodal status, tumor grade, proliferative activity, receptor status and HER2 overexpression, are currently used for determining the risk of relapse of breast cancer patients. Traditional prognostic factors have shown limited ability to predict distinct patient outcomes and individuals with the same clinical assessment can have markedly different courses [32]. Prognostic heterogeneity is complicated by a myriad of liable alterations within multiple biological pathways, stressing the need for further studies on molecular events involved in cancerogenesis, tumor progression and metastasis.

In this study, we explored traditional prognostic factors and a panel of tumor markers not used in routine diagnosis, such as NHERF1, VEGFR1, HIF-1α and TWIST1, that have been respectively related to breast cancer progression [17-19], aggressiveness [21,22], hypoxic response [29,33] and cell invasion/metastasis [24,34], assessing if they are differentially expressed in tumors scored as grade 2, in order to best characterize them. We showed that several parameters discriminated poor versus good clinical outcome on 187 patients, but the PVI together with MIB1 were the two variables that remained strongly associated with worse prognosis in multivariate analysis. Our results are consistent with numerous findings, which highlight proliferation-related genes as the main and common denominator for predicting clinical outcome [35,36]. However, it is not surprising that these genes are involved in breast cancer prognosis, considering that the increased rate of proliferation and defects in cell-cycle regulators are two of the features common to breast carcinomas.

In the present study, overexpression of cytoplasmic NHERF1 resulted associated with unfavorable prognosis and aggressive clinical parameters, as previously demonstrated [18,31]. However, in line with several reports describing the NHERF1 gene as transcriptionally regulated by estrogen [18,31,37], here we demonstrated that cytoplasmic NHERF1 expression was significantly correlated with increasing ER expression. Interestingly, we also observed a significant direct correlation between increased levels of cytoplasmic NHERF1 and VEGFR1. Tumors overexpressing NHERF1 and VEGFR1 revealed an association with poor outcome, being characterized by an increasing tumor grade, and negative status of steroid hormone receptors. It is now clear that NHERF1 promotes dimerization and activation of many tyrosine kinase receptors, such as the platelet derived growth factor receptor [38], the epidermal growth factor receptor [39] and the HER2 [17], known to directly regulate cell pathways related to cancer progression. Previously, we observed that VEGFR1 was expressed in the cytoplasm of breast cancer cells, where also NHERF1 expression was predominant [19]. In this context, NHERF1 acts as a potential interacting partner of VEGFR1, a marker correlated with high metastasis risk and relapse [21,22], probably promoting invasion of tumor cells through autocrine and paracrine mechanisms [40].

In our series, tumors of grade 2 represent 46% of the whole cohort, confirming previous observations that this constitutes a substantial proportion of cases in current routine breast cancer diagnostics [41]. The Elston and Ellis modified grading system provides a simple, inexpensive and routinely applicable overview of the intrinsic biological characteristics and clinical behavior of tumors [4]. Grade 2 tumors usually show an intermediate outcome during the early years of follow-up, leading to speculate the uncertain role of histological grade in therapeutic planning due to the not informative results for clinical decision making [6]. Further sub-classification of grade 2 tumors, possibly into low and high risk categories, would be beneficial to improve prognostic stratification of these patients. Notably, we found that grade 2 tumors exhibited significant correlation with parameters of low-risk significance, such as small tumor size, lower nodal stage and proliferative activity and positive steroid hormone receptor status. Afterwards, we investigated what traditional clinicopathological parameters and new potential markers, such as NHERF1, VEGFR1, HIF-1α and TWIST1, could best characterize grade 2 tumors with poor prognosis. Our results demonstrated that a distinct immunophenotype, PVI/membranous NHERF1, is able to categorize grade 2 tumors into two defined subgroups, which exhibited significantly different prognosis. Interestingly, 72% of grade 2 tumors with the PVI+/membranous NHERF1- expression phenotype were associated to an adverse prognosis. However, a significant high proportion of grade 3 tumors showed the same PVI+/membranous NHERF1- expression phenotype, highlighting that grade 2 tumor subgroup with poor prognosis is regarded as being similar to grade 3 cancers. Relevantly, also in the whole cohort the PVI+/membranous NHERF1- expression phenotype displayed a significant correlation with poor prognosis tumors. Since, according to these results, the PVI+/membranous NHERF1- expression phenotype in grade 2 tumors is a poor prognosis factor, we have analyzed PVI/membranous NHERF1 immunophenotypes in a subgroup of patients stratified as having a family history of breast cancer, a category with high biological malignancy, as previously notified [1,19]. Intriguingly, it was emerged that familial tumors with grade 2 were prevalently associated to the PVI+/membranous NHERF1-expression subset, confirming one more time the prognostic relevance of this tumor immunophenotype.

The pathological significance of the PVI, a marker of tumor with metastatic potential and a predictor of breast cancer outcome, has long been appreciated. In the 9th St Gallen meeting, the presence of vascular invasion was included in the category of relevant prognostic factors in lymph node-negative patients [42]. Efforts to detect early metastatic activity, such as diligent pathological examination of sentinel lymph node biopsies would be complimented by the objective evaluation of vascular invasion status of the primary tumor [43]. Vascular invasion has been recently reported as a histoclinical parameter independently associated with poorer survival inside both basal and triple-negative breast cancer phenotypes [44,45]. However, in a our previous work, it has been demonstrated the predictive significance of the PVI for familial patients with BRCA gene mutation risk [27]. Thus, on the basis of our results, the PVI assessment might provide additional information on disease evolution of grade 2 tumors.

The adaptor protein NHERF1 shows a physiological localization at the plasma membrane, but during breast cancerogenesis progressively loses its apical localization becoming mostly cytoplasmic in no longer polarized tumor cells [17]. In our series, 13% of tumors showed NHERF1 still localized in the plasma membrane, and were positively associated with favorable prognosis parameters, such as low tumor grade, positive PR status, and low proliferative activity. The positive prognostic impact of membranous NHERF1 is in agreement with results obtained from our [17,18] and other laboratories [46], suggesting that NHERF1 might behave either as a tumor suppressor, when it is localized at the plasma membrane, or as an oncogenic protein, when it is shifted to the cytoplasm, depending on its subcellular distribution.

Moreover, in the present study the good prognostic relevance of membranous NHERF1 has been demonstrated both in the whole cohort and in subgroup of grade 2 tumors. From a clinical perspective, the PVI+/membranous NHERF1- expression phenotype could improve the accuracy of predicting clinical outcome for a subgroup of patients. Therefore, the current results indicate that the combination of those two markers may be applicable as predictive markers to select patients for more aggressive treatment and follow-up.

## Conclusions

We showed in this study that cytoplasmic NHERF1 colocalizes with the oncogenic receptor VEGFR1 and their significant correlation suggests new potential implications in breast tumor progression. The PVI results the major variable strongly associated with poor prognosis both in the whole series of invasive cancers and in the grade 2 tumors, improving ability to accurately predict risk of progression. Further, it has been demonstrated the intrinsic biological differences characterizing grade 2 tumors, which represent a combination of two histological subtypes with low or high clinical relevance. In particular, the PVI+/membranous NHERF1- expression immunophenotype identifies a category of grade 2 tumors with the worst prognosis, including patients with a family history of breast cancer. These observations support the idea of the PVI+/membranous NHERF1- expression phenotype as a useful marker for the identification of a subset of grade 2 tumors with clinical high risk of poorer prognosis.

## Competing interests

The authors declare that they have no competing interests.

## Authors' contributions

The project and data analyses were coordinated by Annita Mangia. Pathological and histological assessment of all human samples was performed by Giovanni Simone. The laboratory work and statistical analysis of the data obtained were performed by Andrea Malfettone. The manuscript was written by Andrea Malfettone and Annita Mangia, and its critical revision was provided by Concetta Saponaro. Angelo Paradiso and Giovanni Simone contributed to the strategic plan of the work and to critical revision of the manuscript. All authors have read and approved the final manuscript.

## Pre-publication history

The pre-publication history for this paper can be accessed here:

http://www.biomedcentral.com/1471-2407/12/106/prepub
